# The associations between cognitive function and depressive symptoms among older Chinese population: A cohort study

**DOI:** 10.3389/fpsyt.2023.1081209

**Published:** 2023-04-06

**Authors:** Fan Yang, Mingqi Fu, Qitu Hu, Jing Guo

**Affiliations:** ^1^Department of Health Policy and Management, School of Public Health, Peking University Health Science Center, Beijing, China; ^2^Center for Health Policy and Technology Evaluation, Peking University Health Science Center, Beijing, China; ^3^Center for Social Security Studies, Wuhan University, Wuhan, China; ^4^College of Humanities Education, Inner Mongolia Medical University, Hohhot, China

**Keywords:** cognitive function, cognitive decline, depressive symptoms, leisure activities, cohort study, older adults

## Abstract

With the rapid development of society, population aging has emerged as a significant global challenge. This study aimed to evaluate the impact of baseline cognitive performance, current cognitive function, and cognitive decline on subsequent depressive symptoms. Data were obtained from participants aged 65 years and older in the Chinese Longitudinal Healthy Longevity Survey (CLHLS), Wave 2014–2018. Of the 7,192 participants in Wave 2014, 1,627 were included in the analysis. Multivariate regressions were conducted to estimate the associations between cognitive measures and depressive symptoms. Our results indicated that baseline cognitive function was not associated with subsequent depressive symptoms, but current cognitive function was. Furthermore, participants who experienced significant cognitive decline were more likely to develop depressive symptoms. Covariates, including marital status, economic status, physical activity, and recreational activity, were also associated with subsequent depressive symptoms. These findings suggest that slowing cognitive decline is an effective strategy for preventing depressive symptoms in older adults, promoting their health and wellbeing.

## Introduction

Population aging has become a major challenge worldwide due to the rapid development of society. In China, it is predicted that by 2050, 400 million people will be aged above 65, and 150 million of whom will be aged above 80 (1, 2), thus making it necessary to consider the coming health burden. Besides physical health risks, such as increased incidence of mortality and disabilities (3), mental health status and cognitive problems of older adults are also noteworthy. Mild cognitive impairment (MCI) represents a transitional phase between healthy aging and dementia and affects 10–15% of people aged over 65 years old (4). In addition, a Chinese national survey reported that 4.46% of older adults (aged above 60 years old) met clinical depression, and 35.19% had depressive symptoms (5). Therefore, investigating the current situation of older adults’ cognitive function and depressive symptoms is necessary.

Several factors are related to depressive symptoms (5, 6), and the associations between cognitive function and depressive symptoms have been widely examined by researchers (7, 8). The cognitive vulnerability hypothesis suggests that the cognitive triad is a crucial mechanism that facilitates depressive symptoms (8), and bad cognitive performance could increase the risk of developing depressive symptoms. Formánek et al. (7) reported that the people with the highest risk of cognitive decline are the ones who have increased depressive symptoms during their late life. However, previous studies have some limitations, such as using cross-sectional data (9), which limits their causal inference. For some researchers who used a longitudinal design, they were not sufficient to control the influence of some important covariates, including social engagement, lifestyles (including smoking and drinking), etc., which were reported in previous cross-sectional studies (10, 11). Not only is current cognitive performance associated with depressive symptoms, but cognitive decline is also associated with depressive symptoms (12), and biological evidence has been found in previous studies (13). However, it remains uncertain whether cognitive decline could lead to depressive symptoms.

Education, economic status, and marital status are generally reported to be significantly associated with depressive symptoms (10). High education levels are linked to decreasing the prevalence of depression (14), and better economic status is usually associated with better mental health conditions (15). In addition, married individuals generally have a lower prevalence of depressive symptoms (9). Chronic diseases could also be bidirectional with depressive symptoms (16), along with other diseases (17). Other lifestyles, such as drinking tea, have been reported to reduce the risk of depressive symptoms (18), and leisure activities (i.e., productive, physical, intellectual, recreational, and social activities) are related to mental health outcomes (i.e., depression) when considering the covariates (11). However, there is no agreement on the relationship between types of leisure activities and depressive symptoms. For example, Chao (19) reported that engaging in social activities could reduce the risk of depressive symptoms, while Morgan and Bath argued that social activities have no relationship with depressive symptoms (20). Therefore, to better investigate the association between cognitive function and depressive symptoms, it is necessary to consider various types of leisure activities and important covariates.

Thus, this study aims to assess the effect of baseline cognitive performance, current cognitive function, and significant cognitive change on follow-up depressive symptoms while controlling for necessary covariates. We hypothesize that cognitive functioning and cognitive decline could be linked with depressive symptoms, adjusted for covariates such as education and economic status.

To summarize, the method section will introduce the study design, participants, measures, and statistical methods. The results section will display data and main results. The discussion section will conclude the study with a discussion, and the Conclusion section will summarize the study.

## Methods

### Study design and participants

This study utilized data from the Chinese Longitudinal Healthy Longevity Survey (CLHLS), which began in 1998 as a national survey and is conducted every 2–3 years. The study draws on data from the 2014 and 2018 waves of the CLHLS. A targeted random-sample design was employed to ensure the survey’s representativeness, covering 22 randomly selected provinces in China. Face-to-face interviews were conducted with voluntary participants in sampled counties and cities by trained interviewers, during which various aspects of information were collected, including sociodemographic characteristics, health status, lifestyles, daily activities, social engagement, etc. Participants were required to answer all questions themselves, except those related to objective assessments, such as the Chinese Mini-Mental State Examination (MMSE) and the 10-item Center for Epidemiologic Studies Depression Scale (CES-D-10), which were not allowed to be answered by proxy respondents.

**Figure 1:**
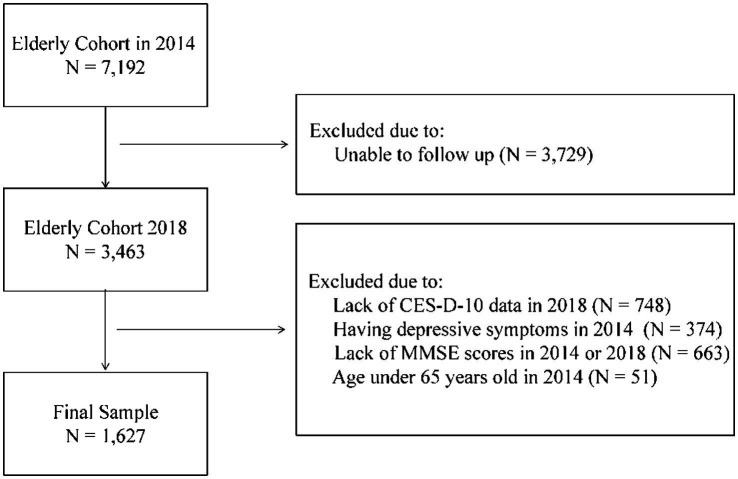
Flow chart of study sample enrollment.

The CLHLS longitudinal data set from 2014 included 7,192 participants aged 65–113 (mean age 78.94, SD 7.79). To achieve the study’s objective, a sample selection procedure was employed (see Figure 1). Participants who had not taken part in the 2018 survey (N = 3,729) due to various reasons, including death, were excluded. Additionally, 748 participants who could not or refused to answer the items of the CES-D-10 in wave 2018 were excluded. Participants (*N* = 374) who were regarded as having depressive symptoms in wave 2014 based on the PhenX Toolkit (PhenX code: 120500) (21) were excluded. PhenX is a short 2-item self-report screening measure that explores whether the participant has had depressive symptoms in the last 12 months lasting 2 weeks or more. Participants were considered depressed if at least one answer was “Yes.” This strict criterion enhanced the study’s ability for causal inference. Furthermore, 663 participants were excluded based on their response to the MMSE, including refusing to answer and missing data (defined as more than 2 of 12 questions not answered properly). Finally, 51 participants under 65 years old were excluded, resulting in 1,627 participants included in the current study. The demographic variables’ contrast between the initial sample and the included sample can be found in the Supplementary material.

## Measures

### Cognitive function

Cognitive function was assessed using the Chinese version of the Mini-Mental State Examination (MMSE), a widely used instrument in Chinese older adults with demonstrated reliability (22). To better reflect the reality of older adults in China and facilitate comprehension and responses, the original MMSE was modified by the Chinese Longitudinal Healthy Longevity Survey (CLHLS). The Chinese MMSE comprises 12 questions assessing five aspects: orientation, registration, attention and calculation, recall, and language, with a total score ranging from 0 to 30. Higher scores indicate better cognitive function. Cognitive decline was defined as a decrease of more than 10% in MMSE scores between wave 2014 and wave 2018 (23). Cronbach’s α was 0.766 in wave 2014 and 0.835 in wave 2018 during the current study.

### Depressive symptoms

Depressive symptoms were assessed using the 10-item Center for Epidemiological Studies Depression Scale (CES-D-10), which has demonstrated validity and reliability among middle-aged and older Chinese adults (24). The CES-D-10 items are rated on a four-point Likert scale ranging from rarely to most of the time (1–2 days to 5–7 days) and have a total score ranging from 0 to 30. A cutoff score of 10 was adapted from a previous study (25) to classify participants into two groups: those with depressive symptoms (score ≥ 10) and those without (score < 10). The Cronbach’s α of CES-D-10 was 0.718 in this study.

### Covariates

We considered socioeconomic characteristics, leisure activities, healthy lifestyles, social engagement, and health conditions at baseline as potential confounding factors, following previous studies (3, 26). Age (continuous), gender (female vs. male), education (no schooling vs. schooling), marital status (married vs. unmarried), residence (rural vs. urban), and self-reported economic status (high, medium, or low, as assessed by the question “How is your living standard compared locally?”) were included as socioeconomic characteristics. Current smoking (yes vs. no) and current alcohol consumption (yes vs. no) were included as indicators of healthy lifestyles.

Leisure activities were divided into five categories: household activities (including housework, garden work, and raising domestic animals), physical activity (including current exercise), intellectual activity (including reading newspapers/books and watching TV and/or listening to radio), recreational activities (including playing cards and/or mahjong), and social activities (including participating in organized social/community activities). Participants were considered to engage in a particular activity if they answered “yes” to the corresponding question.

Activities of daily living (ADLs) were assessed using a scale comprising six questions that covered dressing, eating, toileting, bathing, engaging in indoor activities, and continence. Each activity was scored 1 if it was performed without any assistance and 0 if assistance was required, resulting in an ADL score ranging from 0 to 6. Higher scores indicate better daily function.

Information on hypertension, diabetes, heart disease, cerebrovascular disease, and respiratory disease (asthma and tuberculosis) was collected, and the number of chronic diseases was treated as a continuous variable in our study.

### Data analysis

All analyses were performed using R (version 4.1.2). Continuous variables, including MMSE score, CES-D-10 score, and ADL score, were summarized using mean and standard deviation (SD). Categorical variables such as sex, education, residence, and prevalence of depressive symptoms were presented as frequencies. Logistic regression models were used to examine the relationship between cognitive function and depressive symptoms. The outcome variable was a binary variable indicating the presence of depressive symptoms based on a CES-D score cutoff of 10. Four models were performed. Model 0 included no covariates, while the baseline MMSE score, follow-up MMSE score, and greater cognitive change were added separately. Model 1 included all covariates (age, sex, education, marital status, residence, economic status, current smoking, current alcohol consumption, outdoors activity, household activity, intellectual activity, recreational activity, social activity, ADL score, and number of chronic diseases) to assess the influence of the baseline MMSE score on depressive symptoms. Model 2 added the same covariates as Model 1 to investigate the association between the follow-up MMSE score and depressive symptoms. Model 3 analyzed the association between greater cognitive change and depressive symptoms based on the same covariates as Model 1. A *value of p* <0.05 was considered statistically significant.

### Results

Table 1 presents the characteristics of the study participants at baseline (wave 2014). The mean age of the participants was 78.94 years (SD = 7.79), and 55.56% were female. Participants had a mean number of 0.69 chronic diseases (SD = 0.88). Notably, the mean MMSE score at baseline was 28.07 (SD = 2.55). A total of 242 participants experienced a significant cognitive decline from baseline to follow-up.

**Table 1:** Characteristics of the participants in baseline and follow-up.

Variables	Category	*N*	Mean ± SD/Percentage (%)
Age	/	1,627	78.94 ± 7.79
Gender	Female	904	55.56
	Male	723	44.44
Education	No schooling	686	42.16
	Have schooling	941	57.84
Marital status	Married	791	48.62
	Unmarried	836	51.38
Residence	Rural	805	50.52
	Urban	822	49.48
Economic status	High	306	18.81
	Medium	1,208	74.25
	Low	113	6.95
Current smoking	Yes	328	20.16
	No	1,292	79.84
Current alcohol assumption	Yes	317	19.48
	No	1,295	80.52
Household activity	Yes	1,546	95.02
	No	81	4.98
Physical activity	Yes	561	34.48
	No	1,066	65.52
Intellectual activity	Yes	1,460	89.74
	No	167	10.26
Recreational activity	Yes	561	35.15
	No	1,035	64.85
Social activity	Yes	339	20.84
	No	1,288	79.16
MMSE score	in Wave 2014	1,627	28.07 ± 2.55
	in Wave 2018	1,627	27.49 ± 3.23
MMSE decline	/	1,627	0.58 ± 3.28
CES-D-10 score in wave 2018	/	1,627	5.62 ± 3.68
Depressive symptoms	Have	248	15.24
	Not have	1,379	84.76
ADL score	/	1,573	5.89 ± 0.47
Number of chronic diseases	/	1,604	0.69 ± 0.88

The results of all models in logistic regression are presented in Table 2. In model 0, the baseline MMSE score (OR = 0.95, 95% CI = 0.91–1.00, *p* = 0.049), follow-up MMSE score (OR = 0.93, 95% CI = 0.90–0.96, *p* < 0.001), and MMSE decline (OR = 1.05, 95% CI = 1.01–1.10, *p* = 0.011) were all statistically associated with depressive symptoms. After adjusting for covariates in model 1, the baseline MMSE score was no longer statistically significant (OR = 1.00, 95% CI = 0.94–1.06, *p* = 0.931), while the follow-up MMSE score (OR = 0.95, 95% CI = 0.91–0.99, *p* = 0.019) and MMSE decline (OR = 1.05, 95% CI = 1.00–1.09, *p* = 0.031) remained statistically associated with depressive symptoms.

**Table 2:** Logistic regression for the associations between cognitive function, cognitive change, and depressive symptoms.

Variables	Model 0	Model 1	Model 2	Model 3
	OR (95% CI, *p-value*)	OR (95% CI, *p-value*)	OR (95% CI, *p-value*)	OR (95% CI, *p-value*)
MMSE score in 2014	0.95 (0.91–1.00, *p* = 0.049)	1.00 (0.94–1.06, *p* = 0.931)		
MMSE score in 2018	0.93 (0.90–0.96, *p* < 0.001)		0.95 (0.91–0.99, *p* = 0.019)	
MMSE decline	1.05 (1.01–1.10, *p* = 0.011)			1.05 (1.00–1.09, *p* = 0.031)
Age		1.00 (0.98–1.02, *p* = 0.654)	0.99 (0.97–1.01, *p* = 0.357)	0.99 (0.97–1.01, *p* = 0.560)
Sex				
Male (Ref)				
Female		1.45 (1.04–2.01, p = 0.028)	1.41 (1.01–1.95, *p* = 0.042)	1.44 (1.04–2.01, *p* = 0.028)
Education				
No schooling (Ref)				
Have schooling		0.84 (0.62–1.16, *p* = 0.296)	0.88 (0.64–1.21, *p* = 0.443)	0.86 (0.63–1.19, *p* = 0.370)
Marital status				
Married (Ref)				
Unmarried		1.51 (1.10–2.06, *p* = 0.010)	1.49 (1.09–2.04, *p* = 0.013)	1.50 (1.10–2.06, *p* = 0.011)
Residence				
Urban (Ref)				
Rural		0.86 (0.65–1.15, *p* = 0.320)	0.87 (0.65–1.15, *p* = 0.331)	0.88 (0.66–1.18, *p* = 0.399)
Economic status				
Medium (Ref)				
Low		2.61 (1.69–4.03, *p* < 0.001)	2.65 (1.71–4.09, *p* < 0.001)	2.67 (1.73–4.12, *p* < 0.001)
High		0.56 (0.36–0.88, *p* = 0.011)	0.57 (0.37–0.89, *p* = 0.013)	0.57 (0.37–0.88, *p* = 0.011)
Current smoking				
No (Ref)				
Yes		1.12 (0.75–1.67, *p* = 0.589)	1.10 (0.73–1.65, *p* = 0.642)	1.10 (0.74–1.65, *p* = 0.630)
Current alcohol assumption				
No (Ref)				
Yes		0.99 (0.66–1.47, *p* = 0.953)	1.00 (0.67–1.50, *p* = 0.994)	1.00 (0.67–1.49, *p* = 0.998)
Outdoors activity				
No (Ref)				
Yes		0.64 (0.46–0.89, *p* = 0.008)	0.64 (0.46–0.89, *p* = 0.008)	0.64 (0.46–0.90, *p* = 0.009)
Household activity				
No (Ref)				
Yes		0.87 (0.47–1.60, *p* = 0.645)	0.91 (0.49–1.69, *p* = 0.770)	0.87 (0.47–1.59, *p* = 0.645)
Intellectual activity				
No (Ref)				
Yes		1.04 (0.67–1.61, *p* = 0.869)	1.09 (0.70–1.69, *p* = 0.716)	1.02 (0.66–1.59, *p* = 0.915)
Recreational activity				
No (Ref)				
Yes		0.66 (0.46–0.95, *p* = 0.026)	0.68 (0.47–0.98, *p* = 0.038)	0.67 (0.47–0.97, p = 0.032)
Social activity				
No (Ref)				
Yes		1.08 (0.73–1.59, *p* = 0.698)	1.09 (0.74–1.61, *p* = 0.658)	1.07 (0.73–1.58, p = 0.716)
ADL score		1.06 (0.78–1.43, *p* = 0.719)	1.05 (0.78–1.42, *p* = 0.758)	1.03 (0.77–1.39, *p* = 0.832)
Number of chronic diseases		1.08 (0.92–1.26, *p* = 0.360)	1.08 (0.92–1.26, *p* = 0.351)	1.09 (0.93–1.27, *p* = 0.291)

Mode 0: no covariates included; Model 1, 2. and 3: covariates include age, sex, education, marital status, residence, economic status, current smoking, current alcohol assumption, outdoors activity, household activity, intellectual activity, recreational activity, social activity, ADL score and number of chronic diseases.

Furthermore, the statistical significance of the covariates varied across models, but remained consistent from model 1 to model 3. For example, in model 3, marital status (OR = 1.44, 95% CI = 1.04–2.01, *p* = 0.028), poor economic status (OR = 2.67, 95% CI = 1.73–4.12, *p* < 0.001), outdoor activity (OR = 0.64, 95% CI = 0.46–0.90, *p* = 0.009), and recreational activity (OR = 0.67, 95% CI = 0.47–0.97, *p* = 0.032) were statistically associated with depressive symptoms. However, age, sex, education, current smoking, current alcohol consumption, household activity, intellectual activity, social activity, ADL scores, and number of chronic diseases were not statistically associated with depressive symptoms (*p* > 0.05).

## Discussion

This study aimed to investigate the associations between cognitive function, leisure activities, and depressive symptoms. We found that follow-up cognitive performance and cognitive decline were related to subsequent depressive symptoms, whereas baseline cognitive function showed limited association.

These results are in line with previous studies that have shown a link between worse cognitive function and higher levels of depressive symptoms (8, 9), suggesting that cognitive decline may be a more important factor in the development of depressive symptoms than previous cognitive function (5). These findings are consistent with previous research that late-life depressive symptoms are associated with faster memory decline (27, 28) and that cognitive decline can occur before the onset of depressive symptoms (7). According to LeMoult and Gotlib’s framework (29), cognitive function may contribute to cognitive biases, such as self-referential processing, and cognitive emotion regulation, such as distraction, which could lead to the development of depressive symptoms. Biological factors, such as changes in the function of serum brain-derived neurotrophic factor, may also contribute to these findings (30).

In terms of leisure activities, both physical activity and recreational activity (playing mahjong/cards) showed a statistical association with depressive symptoms. Physical activity may benefit cognitive functioning by reducing the risk of diseases that negatively affect cognition (31) and by improving cardiorespiratory functioning, which contributes to improvements in cognition (31, 32). The quality of leisure activity was also significantly associated with depressive symptoms (11), with recreational activity requiring cooperation and social activities that may improve social relationships and support, leading to a decreased risk of developing depressive symptoms (9).

The links between depressive symptoms, education, economic status, and marital status were widely documented (10, 14). Better education, higher economic status, and better marital status were protective factors for depressive symptoms, with high education levels potentially inhibiting inflammatory cytokine expression and improving cognition to decrease the prevalence of depression (14). Economic status is associated with life conditions and social capital, which could contribute to depressive symptoms (15). Marital status is related to social support, and positive social support can lower the risk of developing depressive symptoms (9). Notably, the number of chronic diseases was also statistically related to depressive symptoms, with physical health and depressive symptoms having a bidirectional relationship (33).

### Limitations, strengths, and implications

Limitations of our study should be noted. First, the self-reported scales used, CES-D-10 and MMSE, may introduce biases compared to clinical diagnosis. Second, bidirectional analysis between cognitive function and depressive symptoms was not possible due to the lack of CES-D-10 scores at baseline. Third, the follow-up rate was not optimal due to the elderly participants. Fourth, the data lacked follow-ups between 2014 and 2018, limiting the assessment of cognitive decline. Fifth, data inefficiency precluded the inclusion of some confounding variables (i.e., behavior of special group (34–36)). Lastly, alternative statistical methods (i.e., mixed-effects models) could be used to provide more comprehensive results.

Therefore, future studies should conduct follow-up studies with shorter intervals, evaluate symptoms by clinical diagnosis based on a larger population with a higher follow-up rate, and use various statistical methods to achieve more comprehensive results.

Despite these limitations, our study has some strengths. First, we based our findings on a national follow-up sample, overcoming the limitation of previous studies based on cross-sectional data. Second, we controlled and discussed the influence of covariates, a limitation mentioned by previous researchers. Third, we excluded participants with potential depressive symptoms at baseline, enhancing the ability of causal inference in our study. Finally, combined with previous studies, we provide evidence that cognitive function may be mutually linked with depressive symptoms.

This study has several implications. First, people, especially the elderly, should pay attention to their cognitive function and take preventive measures if necessary, such as engaging in more physical activity. Second, different leisure activities showed inconsistent associations with depressive symptoms, suggesting physical and recreational activity as potential methods for preventing cognitive decline and depression. Third, our results fit the “successful aging model,” emphasizing the importance of early-life actions, such as regular physical and recreational activity, in successful aging. Finally, our results suggest that cognitive decline may be more important than previous cognitive function, emphasizing the need to prevent cognitive decline regardless of current cognitive function.

## Conclusion

Our findings suggest a positive association between poorer cognitive function and an increased risk of developing depressive symptoms. Furthermore, several covariates, including marital status, economic status, physical activity, and recreational activity, also demonstrated a significant impact on the likelihood of developing subsequent depressive symptoms. These results align with the “successful aging model, “which emphasizes the importance of prevention and intervention strategies for mitigating the onset of depressive symptoms in older adults and promoting overall health and wellbeing.

## Data availability statement

The original contributions presented in the study are publicly available. This data can be found here: https://opendata.pku.edu.cn/dataverse/CHADS. Further inquiries can be directed to the corresponding author/s.

## Ethics statement

The studies involving human participants were reviewed and approved by Research Ethics Committees of Peking University and Duke University for the Protection of Human Subjects (IRB00001052-13074). The patients/participants provided their written informed consent to participate in this study.

## Author contributions

FY and JG drafted the manuscript and analyzed the data. MF and QH revised the manuscript. All authors contributed to the article and approved the submitted version.

## Funding

This work was supported by the International Institute of Population Health, Peking University Health Science Center (Number: JKGL202302), and the National Key Research and Development Plan Project (2022YFC3600904).

## Conflict of interest

The authors declare that the research was conducted in the absence of any commercial or financial relationships that could be construed as a potential conflict of interest.

## Publisher’s note

All claims expressed in this article are solely those of the authors and do not necessarily represent those of their affiliated organizations, or those of the publisher, the editors and the reviewers. Any product that may be evaluated in this article, or claim that may be made by its manufacturer, is not guaranteed or endorsed by the publisher.
