# Supplementary material for: The associations between cognitive function and depressive symptoms among older Chinese population: A cohort study
Source: Front Psychiatry. 2023 Apr 6;14:1081209. doi: 10.3389/fpsyt.2023.1081209 (PMC10117645; doi:10.3389/fpsyt.2023.1081209)
Supplement: Supplementary file 1 [file Table_1.DOCX]

Table S1. The contrast of initial sample and included sample in demographic variables.

| **Variables** | **Levels** | **Selected Sample (N = 1627)** | **Loss to follow-up Sample (N = 5565)** | ***P*-value** |
| --- | --- | --- | --- | --- |
|  |  | **Mean ± SD/ N** | **Mean ± SD/ N** |  |
| Age | / | 78.94 ± 7.79 | 87.18 ± 10.82 | < 0.001 |
| Gender | Female | 904 | 3153 | 0.45 |
|  | Male | 723 | 2412 |  |
| Education | No schooling | 686 | 3335 | < 0.001 |
|  | Have schooling | 941 | 2181 |  |
| Marital status | Married | 791 | 3531 | < 0.001 |
|  | Unmarried | 836 | 1851 |  |
| Residence | Rural | 805 | 3073 | < 0.001 |
|  | Urban | 822 | 2492 |  |
| Economic status | High | 306 | 826 | < 0.001 |
|  | Medium | 1,208 | 3813 |  |
|  | Low | 113 | 768 |  |
